# Prevalence and associated metabolic factors for thyroid nodules: a cross-sectional study in Southwest of China with more than 120 thousand populations

**DOI:** 10.1186/s12902-021-00842-2

**Published:** 2021-08-28

**Authors:** Li Xu, Fanling Zeng, Yutong Wang, Ye Bai, Xuefeng Shan, Lingxi Kong

**Affiliations:** 1 Health Management Centre, The First Affiliated Hospital of Chongqing Medical University, 400016 Chongqing, China; 2 Department of Health Management Centre (Epidemiology and Biostatistics), First Affiliated Hospital, Army Medical University, 400038 Chongqing, China; 3 Department of Epidemiology and Health Statistics, School of Public Health and Management, Chongqing Medical University, 400016 Chongqing, China; 4 Department of Pharmacy, The First Affiliated Hospital of Chongqing Medical University, 400016 Chongqing, China

**Keywords:** Thyroid nodules, Prevalence, Metabolic factors, Multiple thyroid nodules

## Abstract

**Objective:**

To explore the prevalence and its associated metabolic factors of thyroid nodules (TNs) among subjects who participated in the physical examinations in Chongqing, China.

**Methods:**

The participants from the Health Management Center of JinShan Hospital of Chongqing Medical University, between September 2015 and May 2020, were included in this study. All of the participants underwent thyroid ultrasonography, anthropometric measurements, and serum examinations. Differences in the TNs prevalence were compared with the chi-square test or Wilcoxon rang-sum test. Multivariable logistic regression analyses were used to estimate the metabolic factors associated with TNs and multiple thyroid nodules (MTNs).

**Results:**

Of the included 121,702 participants, 41,547 had TNs, and 20,899 had MTNs, with the prevalence of 34.1 and 17.0 %, respectively. Women had a significantly higher prevalence of TNs than men (40.6 % vs. 29.8 %; *χ*^2^ = 1517.33, *P* < 0.001), and TNs prevalence was gradually increased with age (*P* for trend < 0.001). Female gender, advanced age, and metabolic factors including central obesity, hypertension, diabetes and fatty liver were positively associated with TNs; BMI, hyperlipoidemia and hyperuricemia were not independent risk factors of TNs. While female gender, advanced age, central obesity, hypertension and diabetes were independent risk factors of MTNs.

**Conclusions:**

The prevalence of thyroid nodules was relatively high. The associated factors identified in this study could help the clinicians to detect the high-risk patients and make targeted screening strategies for the preventing of the occurrence of TNs.

## Background

Thyroid nodules (TNs), one of the most common thyroid disease, has been defined as discrete lesions within the thyroid gland, radiologically distinct from surrounding thyroid parenchyma [1]. It is reported that the prevalence of TNs was about 22.7 % in China, which may inflict a heavy disease burden on the patients [2]. However, due to the variations in the distributions of age, race and gender composition, and the relatively smaller sample size in previous studies, the reported TNs prevalence was not consistent, ranging from 10 to 50 %, which makes it difficult for drawing a conclusive conclusion [3–7].

TNs thyroid cancer occurs in approximately 7–15 % of thyroid nodules [8]. However, most of the TNs patients are asymptomatic and the increased use of ultrasound (US) allows them to be diagnosed as early as possible. Having a better look at the associated factors for TNs may help the clinicians to identify the high-risk populations and provide preventive treatment and management with them. Previous studies showed metabolic factors, including obesity, hypertension, diabetes, dyslipidemia, and hyperuricemia, and fatty liver were associated with the risk of TNs, which provides opportunities for the identification of high-risk patients [9–14]. But researches based on large samples were still lacked, and the investigations were seldom conducted in China, which partly limited the application of these findings.

In the present study, we aimed to conduct a cross-sectional study incorporating over 120 thousand population in Chongqing, China, to identify the prevalence and metabolic risk factors for TNs and multi thyroid nodules (MTNs).

## Subjects and methods

### Subjects

Participants who participated in the physical examination in the Health Management Center of JinShan Hospital, the First Affiliated Hospital of Chongqing Medical University, between September 2015 and May 2020 were included. During this period, a total of 243,768 subjects underwent general physical examination. After excluding the subjects with multiple medical examinations and the ones: (1) did not receive thyroid ultrasound or abdominal ultrasound(US) examination; (2) people under 18 years old, pregnant or lactating women; (3) with a history of thyroid surgery or drug treatment with thyroid disease; 4)with cancer or other serious illness. In total, 121,702 subjects were included in the final analysis. Our research complies with the Declaration of Helsinki, and the protocol was approved by the Ethics Committee of The First Affiliated Hospital of Chongqing Medical University (2020 − 868). Informed consent has been obtained from each patient according to the requirements of the committee before data collection.

### Anthropometric parameters

Collection of demographic information and medical histories as well as anthropometric examination of the subjects was completed by the trained nurses. Height (m) and weight (kg) was measured by a health analyzer (SK-X80). Body mass index (BMI) was calculated as weight/height^2^. Systolic blood pressure (SBP) and diastolic blood pressure (DBP) were measured in the sitting position with a wrist sphygmomanometer (HBP9020, Omron, Kyoto, Japan). The waist circumference (WC) was measured midway between the lowest ribs and the iliac crest with a folding tape according to the World Health Organization and International Diabetes Federation [15].

### Laboratory examination

For each subject, venous blood samples were taken for laboratory examination after a fasting period of 8 h. Fasting blood glucose (FBG) were detected by the hexokinase method and blood uric acid (UA) was detected by the uricase peroxidase method. Serum triglyceride (TG), total cholesterol (TC), low-density lipoprotein cholesterol (LDL-C), and high-density lipoprotein cholesterol (HDL-C) were detected by the enzymatic method. All the above measurements were done by using an automatic chemistry analyzer (Hitachi 7600, Hitachi Corporation, Tokyo, Japan). Reference ranges of the blood tests were listed as follows: 3.9-6.1mmol/L for FBG,155–357 umol/L for UA, 2.80–5.20 mmol/L for TG, 0.35–1.70 mmol/L for TC, 2.07–3.10 mmol/L for LDL-C and 0.90–1.80 mmol/L for HDL-C.

### Ultrasound Examination

Thyroid Ultrasound Examination: All of the subjects with a supine position fully exposed the neck and then the thyroid ultrasound examination was performed by a specific thyroid sonographer using a 7-MHz linear transducer (PVT-705bt, Toshiba, Japan). For each nodule, size (length, width and depth), shape, location, echogenicity, boundary, and vascularity were collected and recorded.

Abdominal Ultrasound Examination: Abdominal ultrasound examination was performed by a professional sonographer using a 3.5-MHz specific transducer (PVT-375bt, Toshiba, Japan).

### Definition of variables

Thyroid Nodules (TNs): Thyroid nodules was diagnosed according to the 2011 version of thyroid imaging report and data system (TI-RADS) classification criteria for risk assessment of malignant thyroid nodules [16]. Single thyroid nodules (STNs) were defined as only one nodule on any side of thyroid, while multiple thyroid nodules (MTNs) were defined as two or more than two nodules in one or both sides of the thyroid [1].

Fatty Liver: Fatty liver was diagnosed with the US features as follows: (1) Diffuse enhancement of near-field echo of the liver (stronger than that in the kidney), gradual attenuation of the far-field echo; (2) unclear display of intrahepatic lacuna structure; (3) mild to moderate hepatomegaly; (4) unclear display of right lobe of liver and diaphragm [17].

Obesity: According to the Working Group on Obesity in China [18, 19],underweight, normal weight, overweight, and obesity were defined as BMI < 18.5, 18.5–23.9, 24–27.9, and ≥ 28 respectively. Central obesity was defined as waist circumference(WC) ≥ 85 cm for men and ≥ 80 cm for women.

Hypertension: SBP/DBP ≥ 140/90mm Hg (1mm Hg = 0.133 kPa) or patients having been diagnosed with hypertension and receiving treatment [20].

Glucose status: Normal blood glucose and impaired fasting glucose(IFG) were defined as FBG < 6.1mmoLand 6.1mmol/L ≤ FBG < 7.0mmol/L respectively, according to the American Diabetes Association criteria [21]. Diabetes mellitus(DM) was defined as FBG ≥ 7.0mmol/L or under treatment of insulin or glucose-lowering drugs.

Dyslipidemia and Hyperuricemia: Dyslipidemia were classified as hypercholesterolemia(≥ 5.2mmol), hypertriglyceridemia (≥ 1.7 mmol/L), High LDL-C (≥ 3.1mmol/L) and low HDL-C(< 0.9mmol/L), and hyperuricemia was defined as uric acid (UA) > 428 umol/L for men and UA > 357 umol/L for women respectively.

### Statistical Analysis

The SPSS 22.0 software package (IBM, SPSS Inc., USA) was used for statistical analysis. Continuous data were described by mean ± standard deviation (SD); categorical data were described by percentage and counts. Differences in the prevalence of TNs between different groups were evaluated by chi-square test or Wilcoxon rang-sum test. Differences of mean value among individuals with or without TNs were evaluated by unpaired t-test. Univariate binary logistic regression analysis was applied to assess the associations of metabolic factors and TNs and MTNs, respectively. The variables with *p* value < 0.05 were included in multivariable binary logistic regression analysis. All tests were two-tailed, and *p* values < 0.05 were considered of significant significance.

## Results

### Baseline Features of General Population

Of the included 121,702 subjects (73,139 were males and 48,563 were females), 41,547 were found to have TNs, with the prevalence of 34.1 % (Table 1). Among those patients with TNs, STNs accounted for 49.7 % (*N* = 20,648) of the patients, while MTNs accounted for 50.3 % (*N* = 20,899), with the prevalence of 17.0 and 17.2 %, respectively. The prevalence of TNs in women (40.6 %, *N* = 19,734) was significantly higher than that in men (29.8 %, *N* = 21,813) (χ^2^ = 1517.33, *P* < 0.001) (Table 1). The mean age of all the subjects was 42.44 ± 13.24 years, the age of those with TNs (47.89 ± 13.97 years) was older than those without TNs (39.62 ± 11.90 years) (*P* < 0.001) (Table 1). There was a significant difference in age between TNs and those without TNs (W = 1.10 × 10^9^, *P* < 0.001) (Table 1). Furthermore, the prevalence of TNs was found to be increased with age (*P for trend* < 0.001). Of the121,702 healthy subjects, mean BMI was 23.51 ± 4.06 kg/m^2^, mean WC was 81.37 ± 9.95 cm, mean SBP was 123.61 ± 17.30 mmHg, mean DBP was 75.07 ± 11.54 mmHg, mean FBG was 5.43 ± 1.19 mmol/L, mean TC was 4.91 ± 0.94 mmol/L, mean TG was 1.64 ± 1.49 mmol/L, mean HDL-C was 1.40 ± 0.34 mmol/L, mean LDL-C was 2.96 ± 0.81 mmol/L and mean UA was 350.32 ± 95.51 µmol/L(Table 1). Compared with those without TNs, the individuals with TNs had a significantly higher level of BMI, WC, SBP, DBP, FBG, TC, TG, HDL-C and LDL-C (*P* < 0.001) (Table 1).

**Table 1:** Baseline features of general population(*N* = 121,702 )

	Total	TNs	without TNs	χ2/w/t	*P* value
**Total(%)**	121,702	41,547(34.1)	80,155(65.9)		
Female	48,563	19,734(40.6)	28,829(59.4)	1517.33	< 0.001
Male	73,139	21,813(29.8)	51,326(70.2)		
**Age (years)**	42.44 ± 13.24	47.89 ± 13.97	39.62 ± 11.90	-102.924	< 0.001
**Age(years,%)**					
≤ 30	25,455	4912(19.3)	20,543(80.7)	1.10 × 10^9^	< 0.001
31–40	35,672	8527(23.9)	27,145(76.1)		
41–50	27,063	10,080(37.2)	16,983(62.8)		
51–60	20,062	9845(49.1)	10,217(50.9)		
≥ 61	13,450	8183(60.8)	5267(39.2)		
**BMI(Kg/m**^**2**^**)**	23.51 ± 4.06	23.72 ± 3.89	23.40 ± 4.15	-12.477	< 0.001
**WC(cm)**	81.37 ± 9.95	81.87 ± 9.83	81.10 ± 10.00	-12.234	< 0.001
**SBP (mmHg)**	123.61 ± 17.30	126.36 ± 18.58	122.20 ± 16.42	-36.724	< 0.001
**DBP(mmHg)**	75.07 ± 11.54	76.26 ± 11.84	74.45 ± 11.33	-24.5	< 0.001
**FBG(mmol/L)**	5.43 ± 1.19	5.57 ± 1.35	5.36 ± 1.10	-26.95	< 0.001
**TC(mmol/L)**	4.91 ± 0.94	4.99 ± 0.95	4.87 ± 0.93	-20.69	< 0.001
**TG(mmol/L)**	1.64 ± 1.49	1.66 ± 1.48	1.63 ± 1.49	-2.71	< 0.001
**HDL-C(mmol/L)**	1.40 ± 0.34	1.42 ± 0.35	1.39 ± 0.34	-12.8	< 0.001
**LDL-C(mmol/L)**	2.96 ± 0.81	3.01 ± 0.82	2.93 ± 0.81	-15.18	< 0.001
**UA(µmol/L)**	350.32 ± 95.51	340.72 ± 93.52	355.26 ± 96.15	25.21	< 0.001
Female	279.92 ± 62.28	283.38 ± 64.73	277.57 ± 60.45	-9.827	< 0.001
Male	396.3 ± 84.83	391.6 ± 85.34	398.29 ± 84.53	9.71	< 0.001
**Fatty Liver**					
**No**	84,098	27,696(32.9)	56,402(67.1)	146.61	< 0.001
**Yes**	33,976	12,442(36.7)	21,534(63.3)		

*TNs* thyroid nodules; *BMI* body mass index; *WC* waist circumference; *SBP* systolic blood pressure; *DBP* diastolic blood pressure; *FBG* fasting blood glucose; *TC* total cholesterol; *TG* triglycerides; *LDL-C* low-density lipoprotein cholesterol; *HDL-C* high-density lipoprotein cholesterol; *UA* uric acid

### Association Between Metabolic Factors and TNs risk

As shown in Table 2, the univariate binary logistic regression suggested BMI, central obesity, hypertension, higher blood glucose, hypercholesterolemia, hypertriglyceridemia, high level of LDL-C, hyperuricemia and fatty liver were all positively associated with TNs risk, while no association was found in HDL-C (Table 2). When stratified by gender of the participants, all of the metabolic factors, including HDL-C, were associated with the TNs risk (Table 2).

**Table 2:** Association between metabolic factors and thyroid nodules analyzed by univariable binary logistic regression (*N* = 121,702 )

	Total				Male				Female			
	**TNs(N,%)**	**Normal(N,%)**	**OR(95% CI)**	***P*value**	**TNs(N,%)**	**Normal(N,%)**	**OR(95% CI)**	***P *value**	**TNs(N,%)**	**Normal(N,%)**	**OR(95% CI)**	***P *value**
**BMI(Kg/m**^**2**^**)**												
** ≤ 18.4**	1321(26.0)	3751(74.0)	0.71(0.67–0.76)	**< 0.01**	319(21.7)	1152(78.3)	0.73(0.64–0.83)	**< 0.01**	1002(28.1)	2599(71.9)	0.60(0.56–0.65)	**< 0.01**
**18.5–23.9**	19,603(33.1)	39,659(66.9)	1.0(ref)	1.00	8333(27.5)	22,009(72.5)	1.0(ref)	1.00	11,270(39.0)	17,659(61.0)	1.0(ref)	1.00
**24.0-27.9**	13,418(36.5)	23,390(63.5)	1.16(1.13–1.19)	**< 0.01**	9270(32.2)	19,509(67.8)	1.25(1.21–1.30)	**< 0.01**	4148(51.7)	3881(48.3)	1.67(1.59–1.76)	**< 0.01**
**≥ 28.0**	3313(35.7)	5976(64.3)	1.12(1.07–1.17)	**< 0.01**	2418(31.7)	5207(68.3)	1.23(1.16–1.30)	**< 0.01**	895(53.8)	769(46.2)	1.82(1.65–2.01)	**< 0.01**
**Central obesity**												
**No**	19,166(31.4)	41,810(68.6)	1.0(ref)	1.00	7670(25.8)	22,104(74.2)	1.0(ref)	1.00	11,496(36.8)	19,706(63.2)	1.0(ref)	1.00
**Yes**	18,455(37.4)	30,890(62.6)	1.30(1.27–1.34)	**< 0.01**	12,661(33.0)	25,727(67.0)	1.42(1.37–1.47)	**< 0.01**	5794(52.9)	5163(47.1)	1.92(1.84–2.01)	**< 0.01**
**Hypertension**												
**No**	26,954(31.0)	60,094(69.0)	1.0(ref)	1.00	13,406(26.3)	37,593(73.7)	1.0(ref)	1.00	13,548(37.6)	22,501(62.4)	1.0(ref)	1.00
**Yes**	10,829(45.6)	12,933(54.4)	1.87(1.81–1.92)	**< 0.01**	6985(40.2)	10,392(59.8)	1.88(1.82–1.95)	**< 0.01**	3844(60.2)	2541(39.8)	2.51(2.38–2.65)	**< 0.01**
**Glucose status**												
**Normal**	34,700(32.5)	71,915	1.0(ref)	1.00	17,328(27.8)	45,033(72.2)	1.0(ref)	1.00	17,372(39.3)	26,882(60.7)	1.0(ref)	1.00
**IFG**	2324(42.9)	3091(57.1)	1.56(1.47–1.65)	**< 0.01**	1591(38.5)	2539(61.5)	1.63(1.53–1.74)	**< 0.01**	733(57.0)	552(43.0)	2.05(1.84–2.30)	**< 0.01**
**DM**	3446(50.1)	3437(49.9)	2.08(1.98–2.18)	**< 0.01**	2529(46.3)	2937(53.7)	2.24(2.12–2.37)	**< 0.01**	917(64.7)	500(35.3)	2.84(2.54–3.17)	**< 0.01**
**TC(mmol/L)**												
**≤ 5.2**	25,162(32.1)	53,194(67.9)	1.0(ref)	1.00	13,284(28.9)	32,705(71.7)	1.0(ref)	1.00	20,489(63.3)	11,878(36.7)	1.0(ref)	1.00
**> 5.2**	15,464(37.7)	25,606(62.3)	1.28(1.25–1.31)	**< 0.01**	8233(31.4)	18,008(68.6)	1.13(1.09–1.16)	**0.01**	7231(48.8)	7598(51.2)	1.64(1.58–1.71)	**< 0.01**
**TG(mmol/L)**												
**≤ 1.7**	27,586(33.5)	54,778(66.5)	1.0(ref)	1.00	12,307(28.9)	30,276(71.1)	1.0(ref)	1.00	15,279(38.4)	24,502(61.6)	1.0(ref)	1.00
**> 1.7**	13,040(35.2)	24,022(64.8)	1.08(1.05–1.11)	**< 0.01**	9210(31.1)	20,437(68.9)	1.11(1.07–1.15)	**< 0.01**	3830(51.7)	3585(48.3)	1.71(1.63–1.80)	**< 0.01**
**HDL-C(mmol/L)**												
**≥ 0.9**	38,754(34.1)	74,737(65.9)		1.00	20,025(29.7)	47,374(70.3)		1.00	18,729(40.6)	27,363(59.4)		1.00
**< 0.9**	1664(33.5)	3309(66.5)	0.97(0.91–1.03)	0.32	1426(31.9)	3049(68.1)	1.11(1.04–1.18)	**< 0.01**	238(47.8)	260(52.2)	1.34(1.12–1.60)	**< 0.01**
**LDL-C(mmol/L)**												
**≤ 3.1**	23,077(32.6)	47,762(67.4)	1.0(ref)	1.00	11,380(29.1)	27,678(70.9)	1.0(ref)	1.00	11,697(36.8)	20,084(63.2)	1.0(ref)	1.00
**> 3.1**	17,341(36.4)	30,284(63.6)	1.19(1.16–1.21)	**< 0.01**	10,071(30.7)	22,745(69.3)	1.08(1.04–1.11)	**< 0.01**	7270(49.1)	7539(50.9)	1.66(1.59–1.72)	**0.01**
**HUA(µmol/L)**												
**No**	31,978(34.9)	59,717(65.1)	1.0(ref)	1.00	15,165(30.7)	34,237(69.3)	1.0(ref)	1.00	25,480(60.2)	16,813(39.8)	1.0(ref)	1.00
**Yes**	8632(31.1)	19,139(68.9)	0.84(0.82–0.87)	**< 0.01**	6354(27.8)	16,512(72.2)	0.87(0.84–0.90)	**< 0.01**	2278(46.4)	2627(53.6)	1.31(1.24–1.39)	**< 0.01**
**Fatty Liver**												
**No**	27,696(32.9)	56,402(67.1)	1.0(ref)	1.00	12,533(28.3)	31,706(71.7)	1.0(ref)	1.00	15,163(38.0)	24,696(62.0)	1.0(ref)	1.00
**Yes**	12,442(36.7)	21,534(63.3)	1.18(1.15–1.21)	**< 0.01**	8879(32.2)	18,662(67.8)	1.20(1.16–1.24)	**< 0.01**	3563(55.4)	2872(44.6)	2.02(1.92–2.13)	**< 0.01**

*TNs* thyroid nodules; *BMI* body mass index; *TC* total cholesterol; *TG* triglyceride; *HDL-C* high-density lipoprotein cholesterol; *LDL-C *low-density lipoprotein cholesterol; *DM* diabetes mellitus; *IFG* impaired fasting glucose ; *HUA* hyperuricemia

**Fig. 1:**
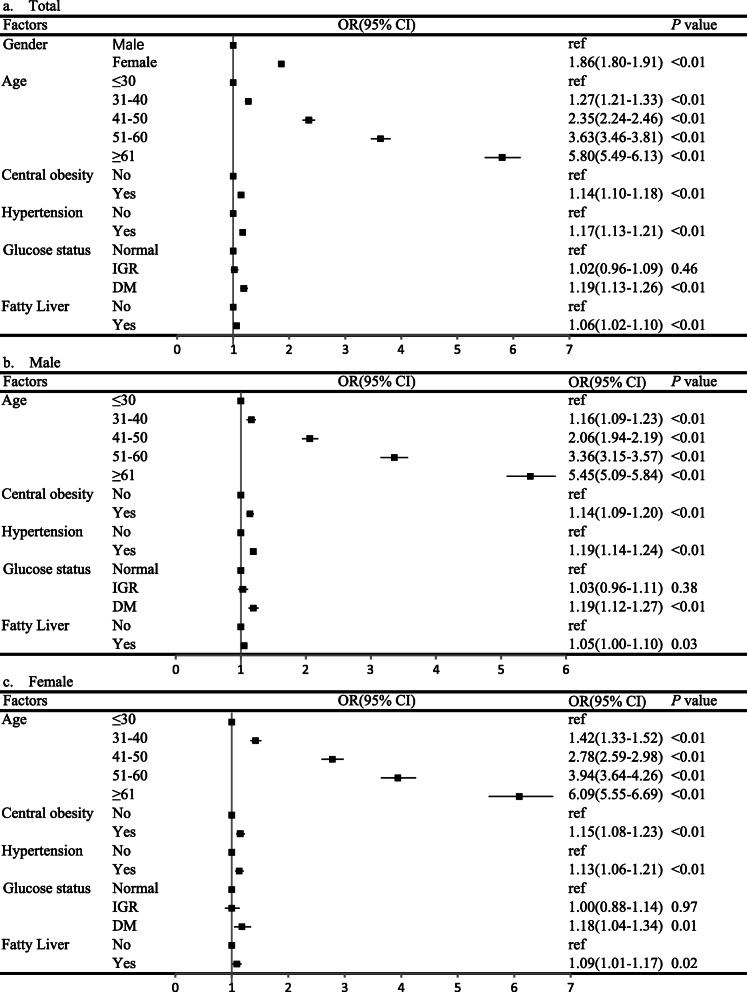
Independent risk factors of thyroid nodules analyzed by multivariate binary logistic regression(*N* = 121,702), adjusted for BMI, TC, TG, HDL-C, LDL-C and HUA. **a**: total population; **b**: male population; **c**: female population. BMI: body mass index; TC: total cholesterol; TG: triglyceride; HDL-C: high-density lipoprotein cholesterol; LDL-C: low-density lipoprotein cholesterol; DM: diabetes mellitus; IFG: impaired fasting glucose; HUA: hyperuricemia

When incorporated all the factors into the multivariable logistic model, results suggested female gender, advanced age, central obesity, hypertension, diabetes, and fatty liver were independently associated with the risk of TNs in the general population (Fig. 1a ). These associations were not significantly changed after sub-grouped by the gender of the participants (Fig. 1b for male and Fig. 1c for female).

### Association Between Metabolic Factors and MTNs compared with STNs

Univariate binary logistic regression analysis of data of 41,547 individuals with TNs indicated that overweight, central obesity, hypertension, high blood glucose, hypercholesterolemia, low level of HDL-C, high level of HDL-C, hyperuricemia, and fatty liver were all significantly associated with the of the prevalence of MTNs compared with STNs, while no association was found between higher TG level and MTNs(Table 3). All of these metabolic factors were associated with the MTNs in females, but for male, overweight, central obesity, hypertension, high blood glucose, low level of HDL-C, high level of LDL-C, and hyperuricemia were associated with the MTNs(Table 3).

**Table 3:** Association between metabolic factors and multiple thyroid nodules analyzed by univariable binary logistic regression(vs. single thyroid nodules, *N* = 41,547 )

			Total				Male				Female	
	**STNs(N,%)**	**MTNs(N,%)**	**OR(95% CI)**	***P*value**	**STNs(N,%)**	**MTNs(N,%)**	**OR(95% CI)**	***P *value**	**STNs(N,%)**	**MTNs(N,%)**	**OR(95% CI)**	***P *value**
**BMI(Kg/m**^**2**^**)**												
** ≤ 18.4**	679(51.4)	642(48.6)	0.96(0.86–1.07)	0.43	163(51.1)	156(48.9)	1.15(0.92–1.44)	0.21	516(51.5)	486(48.5)	0.84(0.73–0.95)	**< 0.01**
**18.5–23.9**	9854(50.3)	9749(49.7)	1.0(ref)	1.00	4555(54.7)	3778(45.3)	1.0(ref)	1.00	5299(47.0)	5971(53.0)	1.0(ref)	1.00
**24.0-27.9**	6497(48.4)	6921(51.6)	1.08(1.03–1.13)	**< 0.01**	4873(52.6)	4397(47.4)	1.09(1.03–1.15)	**< 0.01**	1624(39.2)	2524(60.8)	1.38(1.28–1.48)	**< 0.01**
**≥ 28.0**	1626(49.1)	1687(50.9)	1.05(0.97–1.13)	0.21	1311(54.2)	1107(45.8)	1.02(0.93–1.12)	0.70	315(35.2)	580(64.8)	1.63(1.42–1.88)	**< 0.01**
**Central obesity(cm)**												
**No**	9826(51.3)	9340(48.7)	1.0(ref)	1.00	4278(55.8)	3392(44.2)	1.0(ref)	1.00	5548 (48.3)	5948(51.7)	1.0(ref)	1.00
**Yes**	8813(47.8)	5390(52.2)	1.15(1.11–1.20)	**< 0.01**	6622(52.3)	6039(47.7)	1.15(1.09–1.22)	**< 0.01**	2191(37.8)	3603(62.2)	1.53(1.44–1.64)	**< 0.01**
**Hypertension**												
**No**	14,096(52.3)	12,858(47.7)	1.0(ref)	1.00	7591(56.6)	5815(43.4)	1.0(ref)	1.00	6505(48.0)	7043(52.0)	1.0(ref)	1.00
**Yes**	4626(42.7)	6203(57.3)	1.47(1.41–1.54)	**< 0.01**	3339(47.8)	3646(52.2)	1.43(1.35–1.51)	**< 0.01**	1287(33.5)	2557(66.5)	1.84(1.70–1.98)	**< 0.01**
**Glucose status**												
**Normal**	17,704(51.0)	16,996(49.0)	1.0(ref)	1.00	9614(55.5)	7714(44.5)	1.0(ref)	1.00	8090(46.6)	9289(53.4)	1.0(ref)	1.00
**IFG**	1049(45.1)	1275(54.9)	1.27(1.16–1.38)	**< 0.01**	799(50.2)	792(49.8)	1.24(1.11–1.37)	**< 0.01**	250(34.1)	483(65.9)	1.68(1.44–1.97)	**< 0.01**
**DM**	1392(40.4)	2054(59.6)	1.54(1.43–1.65)	**< 0.01**	1109(43.9)	1420(56.1)	1.60(1.47–1.74)	**< 0.01**	283(30.9)	634(69.1)	1.95(1.69–2.25)	**< 0.01**
**TC(mmol/L)**												
**≤ 5.2**	12,825(51.0)	12,337(49.0)	1.0(ref)	1.00	7099(53.4)	6185(46.6)	1.0(ref)	1.00	5726(48.2)	6152(51.8)	1.0(ref)	1.00
**> 5.2**	7385(47.8)	8079(52.2)	1.14(1.09–1.18)	**< 0.01**	4452(54.1)	3781(45.9)	0.97(0.92–1.03)	0.36	2933(40.6)	4298(59.4)	1.36(1.29–1.45)	**< 0.01**
**TG(mmol/L)**												
**≤ 1.7**	13,697(49.7)	13,889(50.3)	1.0(ref)	1.00	6488(52.7)	5819(47.3)	1.0(ref)	1.00	7209(47.2)	8070(52.8)	1.0(ref)	1.00
**> 1.7**	6513(49.9)	6527(50.1)	0.99(0.95–1.03)	0.58	5063(55.0)	4147(45.0)	0.91(0.87–0.96)	**< 0.01**	1450(37.9)	2380(62.1)	1.47(1.36–1.58)	**< 0.01**
**HDL-C(mmol/L)**												
**≥ 0.9**	19,207(49.6)	19,547(50.4)		1.00	10,733(53.6)	9292(46.4)		1.00	8474(45.2)	10,255(54.8)		1.00
**< 0.9**	884(53.1)	780(46.9)	0.87(0.79–0.96)	**< 0.01**	777(54.5)	649(45.5)	0.96(0.87–1.07)	0.51	107(45.0)	131(55.0)	1.01(0.78–1.31)	0.93
**LDL-C(mmol/L)**												
**≤ 3.1**	11,667(50.6)	11,410(49.4)	1.0(ref)	1.00	6008(52.8)	5372(47.2)	1.0(ref)	1.00	5659(48.4)	6038(51.6)	1.0(ref)	1.00
**> 3.1**	8424(48.6)	8917(51.4)	1.08(1.04–1.13)	**< 0.01**	5502(54.6)	4569(45.4)	0.93(0.88–0.98)	**0.01**	2922(40.2)	4348(59.8)	1.39(1.31–1.48)	**< 0.01**
**HUA(µmol/L)**												
**No**	15,802(49.4)	16,176(50.6)	1.0(ref)	1.00	8059(53.1)	7106(46.9)	1.0(ref)	1.00	7743(46.1)	9070(53.9)	1.0(ref)	1.00
**Yes**	4415(51.1)	4217(48.9)	0.93(0.89–0.98)	**< 0.01**	3495(55.0)	2859(45.0)	0.93(0.87–0.98)	**0.01**	920(40.4)	1358(59.6)	1.26(1.15–1.38)	**< 0.01**
**Fatty Liver**												
**No**	13,884(50.1)	13,812(49.9)	1.0(ref)	1.00	6696(53.4)	5837(46.6)	1.0(ref)	1.00	7188(47.4)	7975(52.6)	1.0(ref)	1.00
**Yes**	6101(49.0)	6341(51.0)	1.04(1.00-1.09)	**0.04**	4785(53.9)	4094(46.1)	0.98(0.93–1.04)	0.50	1316(36.9)	2247(63.1)	1.54(1.43–1.66)	**< 0.01**

*STNs* single thyroid nodules; *MTNs* multiple thyroid nodules; *BMI* body mass index; *TC* total cholesterol; *TG* triglyceride; *HDL-C* high-density lipoprotein cholesterol; *LDL-C* low-density lipoprotein cholesterol; *DM* diabetes mellitus; *IFG* impaired fasting glucose; *HUA* hyperuricemia

**Fig. 2:**
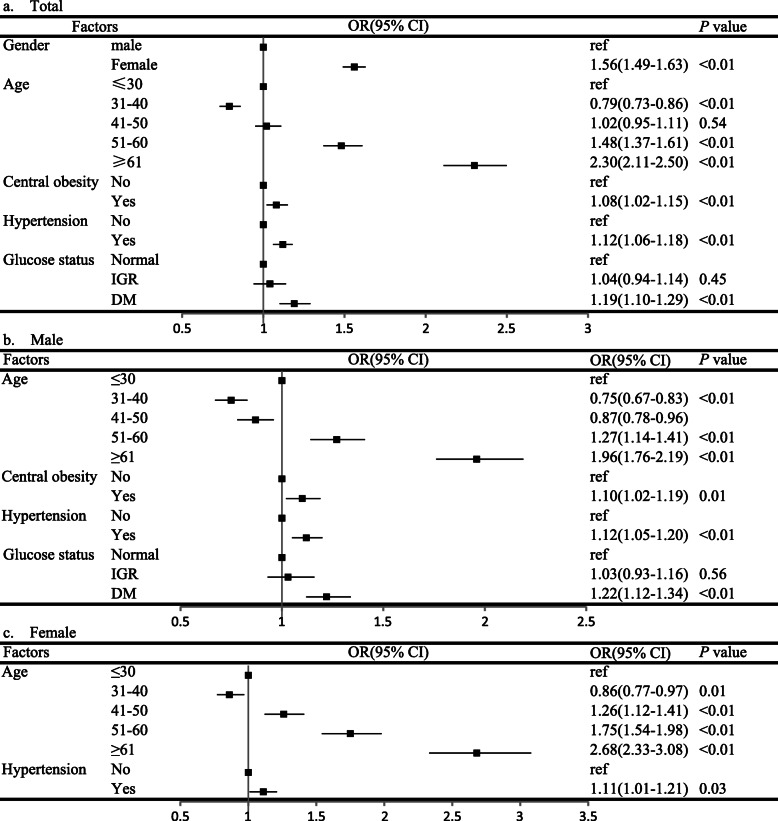
Independent risk factors of multiple thyroid nodules analyzed by multivariate binary logistic regression (vs. single thyroid nodules, *N* = 41,547). **a**: total population, adjusted for BMI, TC, TG, HDL-C, LDL-C, HUA and fatty liver; **b**: male population, adjusted for BMI, TC, TG, HDL-C, LDL-C, HUA and fatty liver; **c**: female population, adjusted for BMI, central obesity, diabetes, TC, TG, HDL-C, LDL-C, HUA and fatty liver. BMI: body mass index; TC: total cholesterol; TG: triglyceride; HDL-C: high-density lipoprotein cholesterol; LDL-C: low-density lipoprotein cholesterol; DM: diabetes mellitus; IFG: impaired fasting glucose; HUA: hyperuricemia

Multivariable binary logistic regression analysis showed that in the general population, female gender, advanced age, central obesity, hypertension and diabetes were independent risk factors of MTNs after adjusting for BMI, level of blood lipids, level of uric acid and fatty liver (Fig. 2a). After stratified by the sex of the participants, advanced age, central obesity, hypertension and diabetes were also associated with the MTNs risk in men, while for female, only advanced age and hypertension were associated with MTNs risk (Fig. 2b for male and Fig. 2c for female)

## Discussion

Thyroid nodule is highly prevalent and is one of the rapidly increasing diseases worldwide. The present study suggested that the prevalence of TNs in Chongqing was 34.1 %, which was lower than the rate reported from the studies in North America (67 %) [22], similar to Germany (33 %) [23] and Kora (34.2 %) [24], and was higher than that in Mexico (19.6 %) [25]. The variations in the TNs prevalence also existed in different regions, even within the same country. During the past ten years, a series of studies tried to investigate the TNs prevalence in China, and a systematic review and meta-analysis also suggested the overall TNs prevalence in China was about 22.7 % [2]. However, studies were seldom conducted in Chongqing city and did not incorporate into previous analyses. The present study suggested the TNs prevalence in Chongqing was relatively higher than the prevalence rate in Guizhou (10.12 %) [26] and Liaoning (10.17 ~ 12.64 %) [27], similar to that in Shandong (31.56 %) [28] and Guangdong (31.65 %) [29], but it was relatively lower than that in Beijing (58.69 %) [30] and Nanjing (46.6 %) [12]. The difference of TNs prevalence between our study and those conducted in the other areas of China may be explained by the difference of lifestyle and dietary habit. Additionally, the prevalence in this study was similar to a recent study reported in Chongqing city, with the prevalence of 32.4 % [31]. Moreover, we also found that the prevalence of MTNs was about 17.0 %, accounted for 50.3 % of the total TNs patients, which is also consistent with the result reported by Liu colleagues [14].

Results of the present study showed that female gender, advanced age and metabolic factors, including central obesity, hypertension, diabetes and fatty liver, were positively associated with the risk of TNs.

Female gender and age have long been proved to be the risk factors of TNs [2, 24, 30, 32]. The gender differences in TNs may be attributed to physiology, pregnancy, and estrogen exposure of female [33]. Estrogen is a substance that promotes the growth of thyroid stem cells and progenitor cells, which contributes to the proliferation of thyroid stem cells through classical genomic and non-genomic pathways and leads to the occurrence of TNs [34, 35]. The results of the present study proved that female population are more likely to be diagnosed with TNs and prevalence of TNs in women and man is 40.6 % vs. 29.8 %, which is similar to that of a study in Shanxi [14]. Increasing age of population is another cause of the high prevalence of TNs, and currently researchers believe that reactive oxygen free radicals precipitate with the rise of age, leading to changes in thyroid tissue and the accumulation of harmful changes in cells [36]. Furthermore, hyperplasia of fibrous connective tissue, inflammatory infiltration, and filtration in the interstitium of thyroid tissue, and vacuolation of the cytoplasm of the vesicle cells eventually result in the formation of nodules in the thyroid tissue [37]. It has been found in our study that the prevalence of TNs increased with age, and the occurrence of TNs reached a high level of 60.8 % in people at the age over 60 years. In other words, ultrasound examination of the thyroid in heath checkups should be highlighted, especially in elder female people.

Strong correlations between body weight, BMI, obesity, waist circumference, and thyroid nodules have been confirmed by some previous studies [8, 11, 14, 24, 38, 39]. It has been reported that the relationship between obesity and thyroid nodules is probably related to leptin secreted by adipose tissue [40]. The increase of serum leptin concentration in the obesity population can promote the rise of thyroid-stimulating hormone levels, which leads to the occurrence and development of thyroid nodules [40]. Results of the present study showed a higher prevalence of TNs in the group of central obesity, and we noticed that in our results BMI of the populations with TNs was higher than that of those without TNs, however, it was not an independent risk factor of thyroid nodules after adjustment. The association between BMI and TNs may depend on different thyroid functions and population grouping methods, which would require further studies for illumination.

Hypertension has been found to be a risk factor of TNs and blood pressure level has been proved to be positively correlated with serum thyroid-stimulating hormone level, which could further increase the prevalence of thyroid nodules [41, 42]. According to previous researches, diabetes might also be related to TNs risk [41] and it has been found that the prevalence of TNs was significantly higher in patients with insulin resistance(IR) than those without IR [43].IR has been reported as an independent risk factor for increased thyroid volume and nodule prevalence, however the exact molecular mechanisms and the pathophysiology were not exactly understood [44–46].It was inferred that IR might cause the proliferation of thyroid cells and promote the formation of nodules and the progression of carcinoma [45]. Additionally, Jornayvaz et al. [47] has found that increase of body fat can be caused by insulin resistance, and with the accumulating and entering of excessive fatty acids in liver, fatty liver forms. Therefore, the onset of fatty liver is related to insulin resistance and fatty liver may be correlated with thyroid nodules through IR. The above findings may explain the higher prevalence of TNs in groups with hypertension, diabetes, and fatty liver in the present study. And it also reminds us that people with hypertension, diabetes and fatty liver should attach high awareness to the occurrence of TNs.

As for the correlation between hyperlipoidemia, uric acid and thyroid nodules, results of our studies proved that they were not independent risk factors of TNs, which was different from that of some previous researchers [9, 14, 32]. Zou et al. [32] reported that high LDL-C was more likely to be associated with MTNs and Yin et al. [9] revealed that elevated triglycerides was a risk factor for TNs, but our data was not consistent with it. In addition, uric acid levels was not associated with the prevalence of TNs after multiple adjustment, which was not in agreement with the results of Liu colleagues [14]. The difference may be possibly explained by the difference of the research subjects enrolled in and the small size of the samples. We believed that more evidence should be needed to clarify the influence of lipids levels and uric acid on TNs.

Besides, the results of the analysis for MTNs indicated that female gender, increasing age, central obesity, hypertension, and diabetes are independent risk factors of MTNs in the general population compared with STNs. When stratified by sex, for men, increasing age, central obesity, hypertension and diabetes are highly associated with the occurrence of MTNs; for women, increasing age and hypertension are independent risk factors of MTNs compared with STNs. Our results suggests that men with central obesity and diabetes should pay more attention to in the diagnosis of MTNs compared with women.

## Conclusions

The present study showed the prevalence of TNs in Chongqing city was as high as 34.1 %. Female gender, advanced age, central obesity, hypertension, diabetes and fatty liver were independent risk factors of TNs, which provides us clues to identify the high-risk patients by a targeted screening strategy of conducting thyroid ultrasonography among women, the elder subjects and those with central obesity, hypertension, diabetes and fatty liver.

## Data Availability

The datasets used and analyzed during the current study available from the corresponding author on reasonable request.

## Abbreviations

STNs: Single thyroid nodules
MTNs: Multiple thyroid nodules
TNs: Thyroid nodules
BMI: Body mass index
WC: Waist circumference
FBG: Fasting blood glucose
IFG: impaired fasting glucose
TG: Triglyceride
TC: Total cholesterol
LDL-C: Low-density lipoprotein cholesterol
HDL-C: High-density lipoprotein cholesterol
US: Ultrasound
UA: Uric acid
TI-RADS: Thyroid imaging reporting and data system
IGR: Impaired glucose regulation
IFG: Impaired fasting blood glucose
DM: Diabetes
IR: Insulin resistance
